# The nuclear factor-κB inhibitor pyrrolidine dithiocarbamate reduces polyinosinic-polycytidilic acid-induced immune response in pregnant rats and the behavioral defects of their adult offspring

**DOI:** 10.1186/1744-9081-7-50

**Published:** 2011-12-31

**Authors:** Xueqin Song, Wenqiang Li, Yongfeng Yang, Jingping Zhao, Chengdi Jiang, Wei Li, Luxian Lv

**Affiliations:** 1 Department of Psychiatry, The First Affiliated Hospital of Zhengzhou University, Zhengzhou, China; 2 Department of Psychiatry, Henan Mental Hospital, The Second Affiliated Hospital of Xinxiang Medical University, Xinxiang, China; 3 Henan Key Lab of Biological Psychiatry, Xinxiang Medical University, Xinxiang, China; 4 The Mental Health Institute of the Second Xiangya Hospital, Central South University, Changsha, China

**Keywords:** cytokine, nuclear factor-kappa B inhibitor, prepulse inhibition, passive avoidance, active avoidance

## Abstract

**Background:**

Epidemiological studies have indicated that maternal infection during pregnancy may lead to a higher incidence of schizophrenia in the offspring. It is assumed that the maternal infection increases the immune response, leading to neurodevelopmental disorders in the offspring. Maternal polyinosinic-polycytidilic acid (PolyI:C) treatment induces a wide range of characteristics in the offspring mimicking some schizophrenia symptoms in humans. These observations are consistent with the neurodevelopmental hypothesis of schizophrenia.

**Methods:**

We examined whether suppression of the maternal immune response could prevent neurodevelopmental disorders in adult offspring. PolyI:C or saline was administered to early pregnant rats to mimic maternal infection, and the maternal immune response represented by tumor necrosis factor alpha (TNF-α) and interleukin-10 (IL-10) levels was determined by enzyme-linked immunosorbent assays (ELISA). The NF-κB inhibitor pyrrolidine dithiocarbamate (PDTC) was used to suppress the maternal immune response. Neurodevelopmental disorders in adult offspring were examined by prepulse inhibition (PPI), passive avoidance, and active avoidance tests.

**Results:**

PolyI:C administration to early pregnant rats led to elevated serum cytokine levels as shown by massive increases in serum TNF-α and IL-10 levels. The adult offspring showed defects in prepulse inhibition, and passive avoidance and active avoidance tests. PDTC intervention in early pregnant rats suppressed cytokine increases and reduced the severity of neurodevelopmental defects in adult offspring.

**Conclusions:**

Our findings suggest that PDTC can suppress the maternal immune response induced by PolyI:C and partially prevent neurodevelopmental disorders of adult offspring.

## Background

Epidemiological studies have indicated that maternal bacterial and viral infections during pregnancy are associated with the emergence of psychosis and related psychopathology in offspring during post-pubescent or adult life [1-3]. Early epidemiological data suggested that maternal infection in the second trimester of human pregnancy conferred the maximum risk for schizophrenia in the offspring [4,5]. However, recent studies have questioned whether the second trimester is exclusively critical [6,7]. Brown et al. [2] showed that infection in the first trimester was also influential. Hence, maternal infections over a more extended period, from early- to mid-pregnancy, can increase the risk of schizophrenia.

However, it is the maternal immune response, rather than direct infection of the fetus, that leads to increased incidence of schizophrenia [8]. Several lines of evidence support this hypothesis [9]. First, in addition to their immunological roles, pro-inflammatory cytokines have various neurodevelopmental effects [10]. Second, increased maternal levels of the pro-inflammatory cytokine tumor necrosis factor-α (TNF-α) and the chemokine interleukin-8 during pregnancy have been directly associated with a higher risk for schizophrenia in the progeny [11,12]. Third, experiments in animals confirm that, in the absence of specific pathogens, prenatal exposure to cytokine-releasing agents [13-18] is sufficient to induce psychopathology in later life. Infection-induced elevation of pro-inflammatory cytokines in the maternal host may be one of the key events leading to enhanced risk of neurodevelopmental disorders in the offspring [19].

Efforts are increasing to develop animal models of schizophrenia. Although attempts to model human psychiatric conditions in animals have always been met with some skepticism, the hypothesized core dysfunctions in schizophrenia are amenable to the development of translational models across species--from mice to human beings. One recently developed model allows the link between maternal immune activation (MIA) and the later development of schizophrenia in offspring to be investigated while separating immune activation from maternal infection [20]. This model uses a single systemic administration of polyinosinic-polycytidilic acid (PolyI:C) to induce MIA in pregnant animals. Systemic exposure to PolyI:C results in an acutely intense elevation of inflammatory cytokines in the host without the production of specific antibodies [20-22]. The offspring of PolyI:C treated dames show largely normal behavior as juveniles [17,23,24]. However, once these animals reach adulthood a number of behavioral features of schizophrenia are evident [18,23-25]. This model is consistent with the neurodevelopmental hypothesis of schizophrenia, which posits that maternal infection provokes an immune response leading to neurodevelopmental disorders in the offspring.

The transcription factor nuclear factor-kappa B (NF-κB) regulates genes involved in cell differentiation, survival/apoptosis, and immune and inflammatory responses [26]. Regulated genes include cytokines, cell surface receptors, and antioxidant enzymes. NF-κB can increase cytokine levels and amplify the inflammation signal of cytokines by the interaction between cytokines and NF-κB in schizophrenia [27]. Here, we examined whether inhibition of NF-κB could suppress the immune response induced by PolyI:C treatment of pregnant rats and thereby reduce neurodevelopmental disorders in the adult offspring.

## Methods

### Chemicals

PolyI:C (potassium salt) and PDTC were obtained from Sigma-Aldrich (Switzerland). PolyI:C was dissolved in phosphate-buffered saline in 5 mg/ml. PDTC was dissolved in physiological saline in 100 mg/ml on the day of injection into rats.

### Animals

Female and male Sprague-Dawley rats were obtained from a specific-pathogen-free (SPF) breeding colony, about ten weeks old, at the Experimental Animal Center of Zhengzhou University (Zhengzhou, China). The rats came from multiple litters. Littermates of the same sex were caged together with four to five per cage. Breeding began after two weeks of acclimation to the new animal holding room. The procedures for breeding and for verification of pregnancy were described by Meyer [13]. All rats were housed in individually ventilated plastic cages at 22 ± 2°C and 50 ± 10% relative humidity with a constant day-night cycle (light: 08:00-20:00 h). Food and tap water were available *ad libitum*. The Animal Care and Use Committee of the Henan Key Lab of Biological Psychiatry (Xinxiang, China) approved the use of rats and the experimental protocols in this study.

### Prenatal treatment

**Figure 1:**
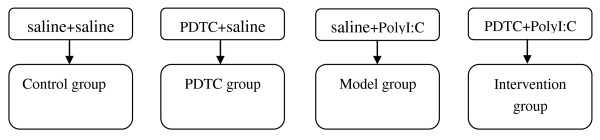
**The treatments of the four experimental groups**.

The rats were mated at an age of about 12 weeks. The first day after copulation was defined as day 1 of pregnancy. Eighty pregnant rats were randomly divided into four groups of 20, designated as intervention, model, PDTC, and control groups. On gestation day 9, the rats in the intervention and model groups were injected with PolyI:C (5 mg/kg) intravenously through the tail vein. The rats in the intervention group received PDTC (100 mg/kg) by intraperitoneal injection 30 min prior to PolyI:C injection, followed by PDTC via intraperitoneal injection and vehicle solution by injection through the tail vein. Those in the model group received intraperitoneal injections of physiological saline vehicle solution instead. The rats in the control group received two intravenous injections of vehicle solution. The treatments of these four experimental groups are showed in Figure 1. In all four groups, half of the rats were retained to fulfill pregnancy; the other rats were executed after injection.

On postnatal day 21, pups were weaned, housed four to a cage by sex and litter, and maintained undisturbed until three months of age. One of each littermates was randomly selected for behavioral testing. The number of subjects employed in the behavioral testing is summarized in Table 1.

**Table 1:** Sample size of each treatment group and experimental condition, and the sequence of behavioral testing

Experiments	Control	PDTC	Prenatal Poly(I:C) treatment(5 mg/kg)	
			
			PDTC (100 mg/kg)	Vehicle
Prepulse inhibition	5♀7♂	6♀5♂	5♀5♂	5♀5♂
Passive avoidance	6♀5♂	5♀6♂	6♀5♂	4♀5♂
Active avoidance procedure	5♀5♂	6♀5♂	5♀5♂	5♀6♂

### Cytokine and NF-κB activation assay

Blood samples were taken from the orbital sinus of rats under methoxyflurane (2,2-dichloro-1,1-difluoro-1-methoxyethane; Pitman-Moore, Titusville, NJ) anesthesia three hours after rats were injected with PolyI:C. Approximately 1 ml of blood per animal was collected. The blood from each animal was separated into serum and white blood cell fractions.

The serum was divided into two parts to permit storage at -80°C until the cytokine assay was performed. IL-10 and TNF-α levels were evaluated using an ELISA kit (R&D Systems) according to the manufacturer's recommendations. The detection limits for IL-10 and TNF-α were 10.0 and 5.0 pg/mL, respectively, with inter-assay variation coefficients of 9.9% and 9.7% and intra-assay variation coefficients of 4.6% and 5.1%, respectively. Standard curve concentrations were calculated in triplicate for each plate.

Nuclear proteins were extracted from peripheral blood mononuclear cells (PBMC) using a Nuclear Extract Kit (Active Motif, Carlsbad, California) according to the manufacturer's instructions. Extracts were stored at 80°C until assayed for the activation of transcription factor NF-κB.

Activation of the NF-κB p65 subunit was determined using an NF-κB p65 ELISA-based transcription factor assay kit (TransAM assay; Active Motif) according to the manufacturer's protocol [28,29]. The NF-κB detection antibody recognizes an epitope on p65 that is accessible only when NF-κB is activated. The detection limit was 0.4 ng recombinant p50 protein per well.

### Prepulse inhibition (PPI) testing

PPI is the normal suppression of a startle response when a low intensity stimulus, which elicits little or no behavioral response, immediately precedes an unexpected stronger startling stimulus. PPI was determined by measuring the decrement in the startle response when the acoustic startle-eliciting stimulus was preceded by an auditory or visual prepulse (PP). The amount of PPI is expressed as the percentage decrease in the amplitude of the startle response caused by presentation of the prepulse.

All test sessions were performed in a single chamber startle apparatus (QMC-I, Kunming Institute of Zoology, Chinese Academy of Sciences, China). One rat was tested during each experimental session. The rat was accommodated in a nonrestrictive Plexiglas cylinder (9 cm diameter, 18.5 cm long) mounted on a floor plate inside a sound- and vibration-attenuating cabinet equipped with a 15 W incandescent bulb and a fan for ventilation. A piezoelectric accelerometer was attached beneath the floor plate to detect and transduce the rat's motor response. A computer program delivered white noise stimuli via an amplifier and a speaker mounted in the chamber above the cylinder. At a rate of 1,000 Hz, the computer sampled accelerometer signals from 200 ms before each acoustic stimulus to 2,300 ms after an acoustic stimulus was delivered. The rats were monitored by a video monitoring system during each test.

In the test session, the rats were acclimated in the testing cylinder for five minutes, during which the rats received only background noise of 70 dB SPL. The test began with six trials of a pulse-alone startle stimulus, consisting of a 40 ms burst of white noise of 120 dB SPL. The session continued with 20 randomized trials, which included five trials of a pulse-alone stimulus and five trials for each of three types of prepulse startles. A prepulse startle consisted of a prepulse (white noise at 2, 4 or 8 dB SPL above the 70 dB SPL background), a 100 ms interval, and a startle pulse (40 ms, 120 dB SPL white noise). The interval length between the 20 randomized trials varied randomly from 8 to 23 s with an average of 15 s.

Startle responses were measured with the program developed by Kunming Institute of Zoology, Chinese Academy of Sciences, China. The peak value of the motor response between auditory stimuli onset and 1,000 ms was automatically analyzed for each trial and the average response was calculated for each type of stimulus. The amount of prepulse inhibition (PPI) was expressed as the percentage decrease in the amplitude of the startle response caused by presentation of the prepulse. The amplitude of the startle response without a prepulse is p. When a weak stimulus is given prior to the startle reflex stimulus, the amplitude for the startle response is pp. The percentage of PPI for each rat was calculated as (1-pp/p) × 100, which is proportional to the inhibitory effect of PPI. Using this description of PPI, a high degree of sensorimotor gating is reflected in a high % PPI value, whereas lower or no gating results in a small or negative % PPI value.

### Passive Avoidance Test

The passive avoidance test is a fear-aggravated test used to assess short-term or long-term memory on small laboratory animals. In this test, subjects learn to avoid an environment in which an aversive stimulus (such as a foot-shock) was previously delivered. The recent memory of the rats was tested in a passive avoidance-conditioning task. Two days after evaluation of general motor activity, learning was evaluated in a single trial, passive avoidance test. The conditioning and testing apparatus consisted of a shuttle box (Ugo Basile model 7550, Comerio, Italy) equipped with a door to restrict access between illuminated and dark compartments of equal size. In the acquisition trial, a rat was placed in the illuminated compartment. After 30 s, the door separating the two compartments was opened. Some seconds later (T1), the animal spontaneously entered the dark compartment. The door was shut 1 s after the crossing, and the rat was given a 0.5 mA, 3 s duration foot shock. Twenty-four hours later (retention trial), the same procedure was repeated without a delay period to open the door and without an electric shock. The elapsed time to enter the dark compartment was recorded as T2.

### Active Avoidance Test

The active avoidance task is a fear-motivated associative avoidance test based on electric current as a source of punishment. This task provides a simple way to assess associative learning and memory of laboratory animals. In a two-way shuttle box apparatus (Panlab, Barcelona), the rats were trained to avoid an aversive unconditioned stimulus (US), an electric shock (0.3 mA) continuously applied to through the floor, associated with the presentation of a light (10 W), which served as a conditioned stimulus (CS). The shuttle box had two compartments (20 × 10 cm) connected by a 3 × 3 cm door. In the compartment containing the mouse, the CS was presented for 5 s followed by concurrent presentation of the CS and US for 25 s. At the end of the 30-s (total) period, the CS and the US were automatically turned off. A conditioned response was recorded when the animal avoided the US by moving to the empty compartment within 5 s of the onset of the CS. If animals failed to avoid the shock, they could escape it by crossing during the US (25 s). Between each trial, there was an interval of 30 s. The ratio of conditioned responses with respect to the total number of changes of compartment was also determined.

The rats received 100 trials per day for 5 consecutive days [30]. Before the start of each session of trials, the rats were placed in the shuttle box for 10 min and allowed to explore. The final rate of active avoidance conditioned response was calculated as (total number of condition responses/500). Higher values indicated better learning and memory.

### Data analysis

Data analyses were conducted using SPSS 13.0 for Windows. Test results were presented as means ± standard deviations (SD). Cytokine data were analyzed using one-way analysis of variance (ANOVA). Active avoidance and passive avoidance test data were analyzed using repeated-measures ANOVA. PPI data were analyzed using multivariate analysis of variance (MANOVA) followed by least significant difference (LSD) post hoc pair-wise comparisons for analysis of differences between groups. Bonferroni corrections were performed for multiple tests.

## Results

Exposure of maternal rats to PolyI:C significantly increased IL-10 and TNF-α protein levels in the maternal serum (Table 2). Treatment effects were evident 3 h after exposure. Intervention with PDTC partially suppressed the increase in cytokine levels. Rats treated only with PDTC showed no effects relative to control-treated rats. NF-κB activation in maternal PBMC is not shown because it was below the detection limit.

**Table 2:** Serum Levels of IL-10 and TNF-α in animals

	Control group	PDTC Group	Model group	Intervention group	*F*	*P*
IL-10(pg/mL)	0.19 ± 0.09^a, b^	0.15 ± 0.09^a, b^	19.14 ± 2.21^b^	13.67 ± 0.97	632.049	< 0.001
TNF-α(pg/mL)	11.96 ± 1.81^a, b^	9.22 ± 3.00^a, b^	128.48 ± 10.38^b^	33.19 ± 2.91	983.570	< 0.001

One-way analysis of variance was used, *P *< 0.05. compared with model group, ^a^*P *< 0.05; compared with intervention group, ^b^*P *< 0.05.

**Figure 2:**
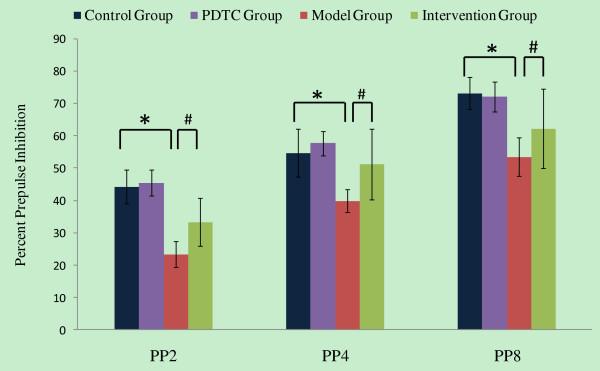
**The effects of PDTC in prenatal PolyI:C administration on PPI of the adult offspring**. PP2, PP4, and PP8 were designated according to the intensity of the prepulse (i.e., 2, 4 or 8 dB SPL above the 70 dB SPL background). Multivariate analysis of variance was used, LSD examination was used for Post hoc analysis, compared with control group, *P < 0.05; compared with intervention group, **^#^***P *< 0.05.

To determine whether adult offspring from mothers with different treatments have PPI defects, PP2, PP4, and PP8 were designated according to the decibel value of prepulse startle stimuli (respectively 2, 4 or 8 dB SPL above the 70 dB SPL background). As expected, the level of prepulse inhibition increased with increasing prepulse intensity for all treatment groups. Multi-factor (treatment×prepulse intensities) ANOVA of %PPI revealed a significant treatment effect (*F *= 58.867, *P *< 0.001) (Figure 2). While the PDTC group did not differ from the control group, offspring from mothers injected with PolyI:C exhibited significantly reduced %PPI relative to controls (*P *< 0.05). The offspring from mothers treated with PolyI:C and PDTC had a higher %PPI (*P *< 0.05) than the rats born to mothers treated with only PolyI:C. Maternal exposure to PolyI:C significantly enhance PPI defects of adult offspring, and this effect can be weakened by treatment with PDTC after exposure.

**Figure 3:**
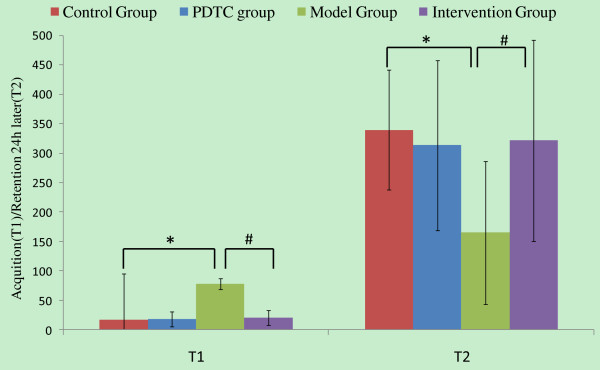
**The effects of PDTC in prenatal PolyI:C administration on passive avoidance behavior of the adult offspring**. Acquisition (T1) and retention 24 h later (T2) were recorded. Multivariate analysis of variance was used, LSD examination was used for Post hoc analysis, compared with control group, *P < 0.05; compared with intervention group, **^#^***P *< 0.05.

PDTC treatment displays significant effect on the performance reduction of the adult offspring from maternal exposure to PolyI:C in a passive avoidance task (Figure 3). ANOVA showed a significant treatment effect (*F *= 135.010, *P *< 0.001). Offspring from the model group (mothers treated with only PolyI:C) had a longer T1 than offspring from the control group (*P *< 0.001), as well as a shorter T2 than offspring from the control group (*P *= 0.04). For the intervention group, this defect was improved from the model group in both T1 (*P *= 0.004) and T2 (*P *= 0.014). These results demonstrate improvements by PDTC treatment on memory impairment in adult offspring from pregnant rats exposed to PolyI:C.

**Figure 4:**
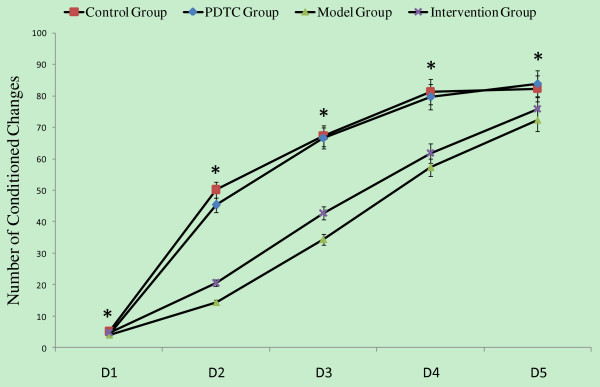
**The effects of PDTC in prenatal PolyI:C administration on active avoidance behavior of the adult offspring**. The average number of conditioned changes in a shuttle box testing apparatus was recorded. Repeated measures ANOVA analysis of variance was used, compared among four groups, *P < 0.05.

Maternal exposure to PolyI:C significantly reduced the performance of adult offspring in an active avoidance task (Figure 4). The offspring showed fewer conditioned responses compared to controls from the first training session. The performance deficit in offspring was reduced by treatment of pregnant rats with PDTC after exposure to PolyI:C. Repeated measures ANOVA revealed a significant main effect of day of training (*F *= 434.264, *P *< 0.001), a significant effect of the treatment (*F *= 17.222, *P *< 0.001), and a significant interaction between these two factors (*F *= 6.934, *P *< 0.001).

Total conditioned response times were significantly different among offspring from the four treatment groups (*F *= 17.22, *P *< 0.001; one-way ANOVA) (Table 3). The average reflex time was higher for the control group than intervention and model groups (*P *< 0.001 for both). No statistical difference was found between intervention group and model group. There were significant differences in total response rates among offspring from the four groups; the rate was higher in offspring from the control group compared to the intervention and model groups (*P *= 0.002, *P *< 0.001). No statistical difference was found between the intervention group and the model group.

**Table 3:** Active avoidance of adult offspring ($\bar{x}\pm s$)

	Control group	PDTC Group	Model group	Intervention group
Total reflex times	285.9 ± 42.36^ab^	279.36 ± 38.19^ab^	182.00 ± 21.97	205.00 ± 52.45
Total reflex rate	0.57 ± 0.085^ab^	0.56 ± 0.076^ab^	0.36 ± 0.043	0.41 ± 0.105

One-way analysis of variance was used, *P *< 0.05. compared with model group, ^a^*P *< 0.05; compared with intervention group, ^b^*P *< 0.05.

## Discussion

Both epidemiological and animal experimental studies have demonstrated that during early pregnancy, maternal immune response mediated by pro-inflammatory cytokines is associated with higher risk for neuropsychiatric disorders in the offspring [31,32]. It was reported that the NF-κB inhibitor, which blocked the NF-κB signaling pathway and reduced cytokine release, was effective in many related diseases [33-35]. In this study, PolyI:C was administered to rats in early pregnancy to stimulate the release of pro-inflammatory cytokines. Maternal cytokine levels and the behavior of adult offspring were measured to explore the role of the cytokine-mediated immune response during pregnancy in the development of psychiatric disorders. We also examined the effects of intervention with an NF-κB inhibitor, PDTC.

In this study, serum levels of IL-10 and TNF-α in the model group (pregnant rats treated with PolyI:C) increased significantly compared to levels in the control group. This imitated inflammatory reactions mediated by cytokines in maternal hosts after infection. Gayle et al. [36] reported that PolyI:C, as well as LPS, could increase the cytokine level in the amniotic fluid and the placenta of the maternal host. Increased cytokines could enter the circulatory system of the fetus.

In our previous study, schizophrenic patients showed activation of NF-κB and elevated levels of cytokines [27]. In the present study, activated NF-κB was below the detection limit in our assays. In the intervention group, NF-κB activation was inhibited through injection of PDTC and serum levels of IL-10 and TNF-α were suppressed relative to the model group. These results provide indirect evidence that NF-κB activation was successfully reduced in the intervention group. All the above indicated that PDTC, a kind of NF-κB inhibitor, can interfere with the inflammatory reactions mediated by cytokines.

Behavioral deficits occurred in offspring from mother rats that had an immune response induced by PolyI:C treatment. The offspring of the model group showed weakened PPI and weakened latent inhibition. These findings are consistent with Meyer [13], who reported that administration of PolyI:C to pregnant mice led to a loss of PPI, loss of latent inhibition, and multiple schizophrenia-like neuropathologic manifestations in the offspring. Behavioral abnormalities were less severe in offspring from the intervention group compared to offspring from the model group, demonstrating that inhibition of NF-κB during pregnancy reduced neurodevelopmental disorders in the offspring.

Latent inhibition exists in all classical and instrumental conditioned reflexes, such as passive and active avoidance. Baruch et al. [37] first reported latent inhibition loss in schizophrenia patients, finding that acute schizophrenic patients lost latent inhibition, while chronic patients treated with antipsychotics presented with normal latent inhibition. Several clinical studies [38-40] further supported this result. Salgado [41] reported that the dopamine antagonist amphetamine could cause loss of latent inhibition in normal healthy people, and conversely, antipsychotics could enhance latent inhibition. Similar results occurred in animals [42]. In addition, individuals from schizophrenic parents showed abnormal latent inhibition. These studies showed that abnormal latent inhibition in patients with schizophrenia could be regarded as a stable manifestation and a cognitive deficit in behavior. In active avoidance tests, the total conditioned reflex time in offspring from the control group was significantly higher than in the offspring from model group (PolyI:C-treated), implying that latent inhibition abnormality, impaired learning, and impaired memory occurred in offspring from model group. In passive avoidance tests, the T1 and T2 in model group offspring were also significantly different from those in control group offspring, indicating that memory was impaired. These results are consistent with previous studies [13-15]. In the active avoidance test, a significant effect of the treatment was found. However, for total conditioned response times, the performance of offspring from the intervention group and model group were not significantly different, demonstrating that NF-κB inhibition did not improve all behavioral outcomes in offspring from the intervention group.

The neurodevelopmental hypothesis of schizophrenia posits a correlation between the disease and neurodevelopmental disorders. It has been suggested that the maternal immune response to viral infections in pregnancy may interfere with normal fetal brain development. Motivated by this hypothesis, researchers have created many animal models to study the effects of prenatal and perinatal environments on schizophrenia. Meyer [13] et al. reported that the PolyI:C treatment model in rats shared a wide range of characteristics with humans, and PolyI:C treatment effects manifested in post-pubescent offspring were consistent with the neural development hypothesis. We demonstrated that prenatal treatment with PolyI:C could elevate maternal cytokines and cause reduced PPI and reduced latent inhibition in adult offspring, confirming Meyer's results. Current studies concentrate on cytokines as a neurodevelopmental disorder trigger in maternal hosts after infection. Pro-inflammatory cytokines released by the maternal immune system may disrupt fetal brain development. Transfer of maternal cytokines to fetuses is not the only means of elevating cytokine levels in fetal brains [43]; the response of fetal immune systems to increased maternal cytokines might be an alternate mechanism [43]. The influence of enhanced anti-inflammatory cytokine signaling on early brain development should be also emphasized [44]. Disruption of the balance between pro- and anti-inflammatory cytokine signaling in fetal brains may be a key mechanism precipitating schizophrenia-related pathology following prenatal maternal infection [45]. In our study, the NF-κB inhibitor in the intervention group evidently suppressed cytokine release induced by PolyI:C and improved behavioral outcomes in adult offspring. The results suggest cytokines play an important role in neurodevelopmental disorders in this model, and provide evidence for the correlation between increased cytokines in maternal hosts and abnormal behavior of offspring. Activated NF-κB was not detected in this study. Thus, there is no direct evidence of a relationship between NF-κB activity and abnormal behavior of offspring. The exact mechanism needs further study.

## Conclusions

Our findings suggest that PDTC treatment during pregnancy can partially reduce neurodevelopmental disorders of adult offspring by suppressing the maternal immune response induced by PolyI:C.

## List of abbreviations

PolyI:C: polyinosinic-polycytidilic acid; TNF-α: necrosis factor alpha; IL-10: interleukin-10; ELISA: enzyme-linked immunosorbent assay; PDTC: pyrrolidinedithiocarbamate; PPI: prepulse inhibition; MIA: maternal immune activation; NF-κB: Nuclear factor-kappa B; US: unconditioned stimulus; CS: conditioned stimulus; LSD: least significant difference; PBMC: peripheral blood mononuclear cell; PPI: prepulse inhibition.

## Competing interests

The authors declare that they have no competing interests.

## Authors' contributions

LL and XS participated in the design of the study and made final approval of the version to be published. XS and WL were involved in drafting the manuscript and data analysis. CJ, YY and WL carried out the animal experiment JZ undertook revise the manuscript. All authors read and approved the final manuscript.

## References

[B1] Brown AS Schaefer CA Wyatt RJ Goetz R Begg MD Gorman JM Susser ES Maternal exposure to respiratory infections and adult schizophrenia spectrum disorders: a prospective birth cohort study Schizophr Bull 2000 26 2 287 95 10885631 10.1093/oxfordjournals.schbul.a033453

[B2] Brown AS Begg MD Gravenstein S Schaefer CA Wyatt RJ Bresnahan M Babulas VP Susser ES Serologic evidence of prenatal influenza in the etiology of schizophrenia Arch Gen Psychiatry 2004 61 8 774 80 10.1001/archpsyc.61.8.774 15289276

[B3] Hultman CM Sparén P Takei N Murray RM Cnattingius S Prenatal and perinatal risk factors for schizophrenia, affective psychosis, and reactive psychosis of early onset: case-control study BMJ 1999 318 7181 421 6 10.1136/bmj.318.7181.421 9974454 PMC27730

[B4] Cannon M Clarke MC Risk for schizophrenia -- broadening the concepts, pushing back the boundaries Schizophr Res 2005 79 1 5 13 10.1016/j.schres.2005.05.027 16005613

[B5] McDonald C Murray RM Early and late environmental risk factors for schizophrenia Brain Res Brain Res Rev 2000 31 2-3 130 7 10719141 10.1016/s0165-0173(99)00030-2

[B6] Mino Y Oshima I Tsuda T Okagami K No relationship between schizophrenic birth and influenza epidemics in Japan J Psychiatr Res 2000 34 2 133 8 10.1016/S0022-3956(00)00003-0 10758255

[B7] Morgan V Castle D Page A Fazio S Gurrin L Burton P Montgomery P Jablensky A Influenza epidemics and incidence of schizophrenia, affective disorders and mental retardation in Western Australia: no evidence of a major effect Schizophr Res 1997 26 1 25 39 10.1016/S0920-9964(97)00033-9 9376335

[B8] Patterson PH Maternal infection: window on neuroimmune interactions in fetal brain development and mental illness Curr Opin Neurobiol 2002 12 1 115 8 10.1016/S0959-4388(02)00299-4 11861174

[B9] Smith SE Li J Garbett K Mirnics K Patterson PH Maternal Immune Activation Alters Fetal Brain Development through Interleukin-6 J Neurosci 2007 27 40 10695 702 10.1523/JNEUROSCI.2178-07.2007 17913903 PMC2387067

[B10] Gilmore JH Fredrik Jarskog L Vadlamudi S Lauder JM Prenatal infection and risk for schizophrenia: IL-1beta, IL-6, and TNFalpha inhibit cortical neuron dendrite development Neuropsychopharmacology 2004 29 7 1221 9 10.1038/sj.npp.1300446 15085088

[B11] Brown AS Hooton J Schaefer CA Zhang H Petkova E Babulas V Perrin M Gorman JM Susser ES Elevated maternal interleukin-8 levels and risk of schizophrenia in adult offspring Am J Psychiatry 2004 161 5 889 95 10.1176/appi.ajp.161.5.889 15121655

[B12] Buka SL Tsuang MT Torrey EF Klebanoff MA Wagner RL Yolken RH Maternal cytokine levels during pregnancy and adult psychosis Brain Behav Immun 2001 15 4 411 20 10.1006/brbi.2001.0644 11782107

[B13] Meyer U Feldon J Schedlowski M Yee BK Towards an immuno-precipitated neurodevelopmental animal model of schizophrenia Neurosci Biobehav Rev 2005 29 6 913 47 10.1016/j.neubiorev.2004.10.012 15964075

[B14] Meyer U Feldon J Schedlowski M Yee BK Immunological stress at the maternal-foetal interface: A link between neurodevelopment and adult psychopathology Brain Behav Immun 2006 20 4 378 88 10.1016/j.bbi.2005.11.003 16378711

[B15] Meyer U Nyffeler M Engler A Urwyler A Schedlowski M Knuesel I Yee BK Feldon J The time of prenatal immune challenge determines the specificity of inflammation-mediated brain and behavioral pathology J Neurosci 2006 26 18 4752 62 10.1523/JNEUROSCI.0099-06.2006 16672647 PMC6674174

[B16] Meyer U Schwendener S Feldon J Yee BK Prenatal and postnatal maternal contributions in the infection model of schizophrenia Exp Brain Res 2006 173 2 243 57 10.1007/s00221-006-0419-5 16552558

[B17] Ozawa K Hashimoto K Kishimoto T Shimizu E Ishikura H Iyo M Immune activation during pregnancy in mice leads to dopaminergic hyperfunction and cognitive impairment in the offspring: a neurodevelopmental animal model of schizophrenia Biol Psychiatry 2006 59 6 546 54 10.1016/j.biopsych.2005.07.031 16256957

[B18] Zuckerman L Weiner I Maternal immune activation leads to behavioral and pharmacological changes in the adult offspring J Psychiatr Res 2005 39 3 311 23 10.1016/j.jpsychires.2004.08.008 15725430

[B19] Gilmore JH Jarskog LF Exposure to infection and brain development: cytokines in the pathogenesis of schizophrenia Schizophr Res 1997 24 3 365 7 10.1016/S0920-9964(96)00123-5 9134598

[B20] Alexopoulou L Holt AC Medzhitov R Flavell RA Recognition of double-stranded RNA and activation of NF-[kappa]B by Toll-like receptor 3 Nature 2001 413 6857 732 8 10.1038/35099560 11607032

[B21] Fortier ME Kent S Ashdown H Poole S Boksa P Luheshi GN The viral mimic, polyinosinic:polycytidylic acid, induces fever in rats via an interleukin-1-dependent mechanism Am J Physiol Regul Integr Comp Physiol 2004 287 4 R759 66 10.1152/ajpregu.00293.2004 15205185

[B22] Traynor TR Majde JA Bohnet SG Krueger JM Intratracheal double-stranded RNA plus interferon-gamma: a model for analysis of the acute phase response to respiratory viral infections Life Sci 2004 74 20 2563 76 10.1016/j.lfs.2003.10.010 15010266

[B23] Zuckerman L Rehavi M Nachman R Weiner I Immune activation during pregnancy in rats leads to a postpubertal emergence of disrupted latent inhibition, dopaminergic hyperfunction, and altered limbic morphology in the offspring: a novel neurodevelopmental model of schizophrenia Neuropsychopharmacology 2003 28 10 1778 89 10.1038/sj.npp.1300248 12865897

[B24] Zuckerman L Weiner I Post-pubertal emergence of disrupted latent inhibition following prenatal immune activation Psychopharmacology (Berl) 2003 169 3-4 308 13 10.1007/s00213-003-1461-7 12748757

[B25] Shi L Fatemi SH Sidwell RW Patterson PH Maternal influenza infection causes marked behavioral and pharmacological changes in the offspring J Neurosci 2003 23 1 297 302 12514227 10.1523/JNEUROSCI.23-01-00297.2003 PMC6742135

[B26] Baeuerle PA The inducible transcription activator NF-kappa B: regulation by distinct protein subunits Biochim Biophys Acta 1991 1072 1 63 80 2018779 10.1016/0304-419x(91)90007-8

[B27] Song XQ Lv LX Li WQ Hao YH Zhao JP The Interaction of Nuclear Factor-Kappa B and Cytokines Is Associated with Schizophrenia Biol Psychiatry 2009 65 6 481 8 10.1016/j.biopsych.2008.10.018 19058794

[B28] O'Hara AM O'Regan P Fanning A O'Mahony C Macsharry J Lyons A Bienenstock J O'Mahony L Shanahan F Functional modulation of human intestinal epithelial cell responses by Bifidobacterium infantis and Lactobacillus salivarius Immunology 2006 118 2 202 15 10.1111/j.1365-2567.2006.02358.x 16771855 PMC1782284

[B29] Renard P Ernest I Houbion A Art M Le Calvez H Raes M Remacle J Development of a sensitive multi-well colorimetric assay for active NFκB Nucleic Acids Res 2001 29 4 E21 10.1093/nar/29.4.e21 11160941 PMC29628

[B30] Trigo JM Cabrero-Castel A Berrendero F Maldonado R Robledo P MDMA modifies active avoidance learning and recall in mice Psychopharmacology (Berl) 2008 197 3 391 400 10.1007/s00213-007-1045-z 18185919

[B31] Meyer U Nyffeler M Schwendener S Knuesel I Yee BK Feldon J Relative prenatal and postnatal maternal contributions to schizophrenia-related neurochemical dysfunction after in utero immune challenge Neuropsychopharmacology 2008 33 2 441 56 10.1038/sj.npp.1301413 17443130

[B32] Meyer U Nyffeler M Yee BK Knuesel I Feldon J Adult brain and behavioral pathological markers of prenatal immune challenge during early/middle and late fetal development in mice Brain Behav Immun 2008 22 4 469 86 10.1016/j.bbi.2007.09.012 18023140

[B33] Loukili N Rosenblatt-Velin N Rolli J Levrand S Feihl F Waeber B Pacher P Liaudet L Oxidants positively or negatively regulate nuclear factor kappaB in a context-dependent manner J Biol Chem 2010 285 21 15746 52 10.1074/jbc.M110.103259 20299457 PMC2871441

[B34] Seymour EM Bennink MR Watts SW Bolling SF Whole grape intake impacts cardiac peroxisome proliferator-activated receptor and nuclear factor kappaB activity and cytokine expression in rats with diastolic dysfunction Hypertension 2010 55 5 1179 85 10.1161/HYPERTENSIONAHA.109.149393 20231522 PMC2929369

[B35] Wang S Quang Le T Chida J Cisse Y Yano M Kido H Mechanisms of matrix metalloproteinase-9 upregulation and tissue destruction in various organs in influenza A virus infection J Med Invest 2010 57 1-2 26 34 20299740 10.2152/jmi.57.26

[B36] Gayle DA Beloosesky R Desai M Amidi F Nuñez SE Ross MG Maternal LPS induces cytokines in the amniotic fluid and corticotropin releasing hormone in the fetal rat brain Am J Physiol Regul Integr Comp Physiol 2004 86 6 R1024 9 10.1152/ajpregu.00664.2003 14988088

[B37] Baruch I Hemsley DR Gray JA Differential performance of acute and chronic schizophrenics in a latent inhibition task J. Nerv Ment Dis 1988 176 10 598 606 10.1097/00005053-198810000-00004 2903219

[B38] Gray NS Snowden RJ The relevance of irrelevance to schizophrenia Neurosci Biobehav Rev 2005 29 6 989 99 10.1016/j.neubiorev.2005.01.006 15967503

[B39] Lubow RE Construct validity of the animal latent inhibition model of selective attention deficits in schizophrenia Schizophr Bull 2005 31 1 139 53 10.1093/schbul/sbi005 15888432

[B40] Yogev H Sirota P Gutman Y Hadar U Latent inhibition and overswitching in schizophrenia Schizophr Bull 2004 30 4 713 26 15954186 10.1093/oxfordjournals.schbul.a007125

[B41] Salgado JV Hetem LA Vidal M Graeff FG Danion JM Sandner G Reduction of latent inhibition by D-amphetamine in a conditioned suppression paradigm in humans Behav Brain Res 2000 117 1-2 61 7 10.1016/S0166-4328(00)00279-5 11099758

[B42] Lubow RE De la Casa G Latent inhibition as a function of schizotypality and gender: implications for schizophrenia Biol Psychol 2002 59 1 69 86 10.1016/S0301-0511(01)00124-7 11790444

[B43] Saviouk V Chow EW Bassett AS Brzustowicz LM Tumor necrosis factor promoter haplotype associated with schizophrenia reveals a linked locus on 1q44 Mol Psychiatry 2005 10 4 375 83 10.1038/sj.mp.4001582 15340354 PMC3133762

[B44] Meyer U Feldon J Yee BK A review of the fetal brain cytokine imbalance hypothesis of schizophrenia Schizophr Bull 2009 35 5 959 72 10.1093/schbul/sbn022 18408229 PMC2728807

[B45] J Haddad JJ Saadé NE Safieh-Garabedian B Cytokines and neuro-immune-endocrine interactions: a role for the hypothalamic-pituitary-adrenal revolving axis J Neuroimmunol 2002 133 1-2 1 19 10.1016/S0165-5728(02)00357-0 12446003

